# Systematic drug repositioning through mining adverse event data in ClinicalTrials.gov

**DOI:** 10.7717/peerj.3154

**Published:** 2017-03-23

**Authors:** Eric Wen Su, Todd M. Sanger

**Affiliations:** Advanced Analytics Hub, Eli Lilly and Company, Indianapolis, IN, United States of America

**Keywords:** Drug repositioning, Drug repurposing, Indication discovery, ClinicalTrials.gov, Text mining

## Abstract

Drug repositioning (i.e., drug repurposing) is the process of discovering new uses for marketed drugs. Historically, such discoveries were serendipitous. However, the rapid growth in electronic clinical data and text mining tools makes it feasible to systematically identify drugs with the potential to be repurposed. Described here is a novel method of drug repositioning by mining ClinicalTrials.gov. The text mining tools I2E (Linguamatics) and PolyAnalyst (Megaputer) were utilized. An I2E query extracts “Serious Adverse Events” (SAE) data from randomized trials in ClinicalTrials.gov. Through a statistical algorithm, a PolyAnalyst workflow ranks the drugs where the treatment arm has fewer predefined SAEs than the control arm, indicating that potentially the drug is reducing the level of SAE. Hypotheses could then be generated for the new use of these drugs based on the predefined SAE that is indicative of disease (for example, cancer).

## Introduction

Drug repositioning (i.e., drug repurposing) involves the identification and development of new uses for existing drugs (Ashburn & Thor, 2004). The best known example of drug repositioning is the serendipitous discovery of the additional use of thalidomide for the treatment of painful sores associated with leprosy. In 1964, Dr. Jacob Sheskin used thalidomide to help a patient sleep, unexpectedly, the thalidomide also healed the patient’s sores and eliminated his pain (Ashburn & Thor, 2004; Sheskin, 1965). This discovery shows that clinical data could be the most direct and reliable source of drug repositioning.

However, systematic drug repositioning efforts since 1964 have not been based on clinical data. Typical approaches include high-throughput screening of marketed drugs (Qosa et al., 2016), targeted testing of a class of drugs for a new disease area (Wu et al., 2016a), and *in silico* methods (Hodos et al., 2016; Mullen et al., 2016), usually based on drug-target interactions (Coelho, Arrais & Oliveira, 2016; Zheng et al., 2015).

Described here is a novel approach to drug repositioning using data from randomized clinical trials. Text mining tools have been used to extract serious adverse event (SAE) data, identify drugs with fewer events related to diseases or associated symptoms in the drug arm than in the control arm, and rank the drugs based on the *z*-score of log odds ratio.

## Materials & Methods

A text mining query was developed to extract SAE data from clinical trial data posted at ClinicalTrials.gov. ClinicalTrials.gov is a registry of federally and privately funded clinical trials conducted in the United States and around the world, and contains rich biomedical data from over 220,000 studies in 191 countries. The query was built using Linguamatics’ I2E, a literature text mining tool based on natural language processing and linguistic analytics (Cormack et al., 2015; Galijatovic-Idrizbegovic et al., 2016).

**Figure 1:**
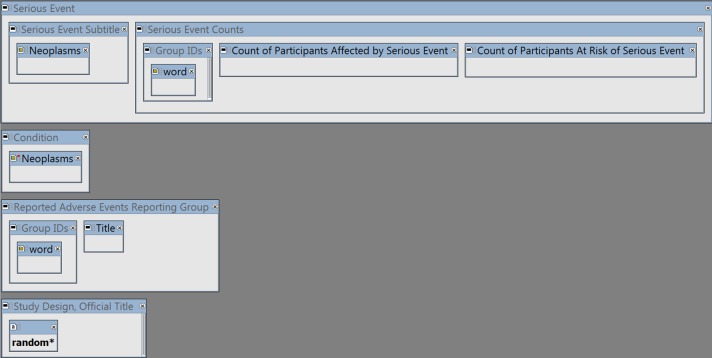
The I2E query. See Supplemental Information 1 to reproduce the query by copying and pasting the YAML script into the I2E Pro interface. The query was run on the I2E index that covers the data posted in ClinicalTrials.gov up to August 14, 2016.

The query (shown in Fig. 1) has 4 main elements:

• To extract Serious Adverse Events classified as cancerous, the combined cancer terms and synonyms from MeSH (https://www.nlm.nih.gov/mesh/) and NCI (http://www.cancer.gov/research/resources/terminology) were loaded into the query region “Serious Event Subtitle” of ClinicalTrials.gov (the “Neoplasms” class).
• The same “Neoplasms” class was negated in the “Condition” region to exclude cancer trials.
• To link the SAE counts to the relevant study arm (i.e., drug or placebo etc.), the group (study arm) IDs and description (“Title”) were extracted from the Reporting Groups region.
• The wildcard “random*” was required in the Study Design or Official Title region to ensure that only randomized trials are reported.

The Excel output from the I2E query in Fig. 1 was loaded into PolyAnalyst (Megaputer) for reformatting and calculating the odds ratios (OR) and *z*-score. The final table was sorted by *z*-score. PolyAnalyst is a commercial text mining tool. The specific tasks described here could also be accomplished by an open-source tool such as KNIME, R, or Python.

The formula for calculating odds ratio (*OR*), standard error (*SE*), 95% confidence interval lower and upper limits (*LowerLimit* and *UpperLimit*), and *z*-score are as follows:

Let *Cs* = Number of patients with **S**AE in **C**ontrol arm; *Cn* = **N**umber of patients in **C**ontrol arm and *Ds* = Number of patients with **S**AE in **D**rug arm; *Dn* = **N**umber of patients in **D**rug arm.

\begin{eqnarray*}OR= \frac{Ds/(Dn-Ds)}{Cs/(Cn-Cs)} \end{eqnarray*}

The distribution of log(*OR*) is approximately normal with:

\begin{eqnarray*}& & SE=\sqrt{ \frac{1}{Cs} + \frac{1}{Cn-Cs} + \frac{1}{Ds} + \frac{1}{Dn-Ds} }\vskip{10pc} \end{eqnarray*}

\begin{eqnarray*}& & LowerLimit=\exp \nolimits (\log \nolimits (OR)-1.96SE) \end{eqnarray*}

\begin{eqnarray*}& & UpperLimit=\exp \nolimits (\log \nolimits (OR)+1.96SE) \end{eqnarray*}

The null hypothesis is that there is no difference between drug and control arm (expected mean OR = 1). Therefore,

\begin{eqnarray*}z= \frac{\log \nolimits (OR)-\log \nolimits (1)}{SE} \text{or}z=\log \nolimits (OR)/SE \end{eqnarray*}

Since the *Cs* and *Ds* are usually small, SE, lower and upper limits, and *z*-score may not be meaningful for hypothesis testing. However, *z*-scores are still useful to rank drugs for hypothesis generation on drug repurposing.

Also because of the multiple comparison nature of the algorithm, the results should only be used for hypothesis generation, not for making any conclusions.

For drugs with *z*-scores ≤ − 1.96, we reviewed the biomedical literature on the drugs, the drug targets, and the disease pathways to see if the hypothesis is consistent with the current scientific knowledge. The literature review was performed using the text mining tool I2E (Bandy, Milward & McQuay, 2009).

## Results

**Figure 2:**
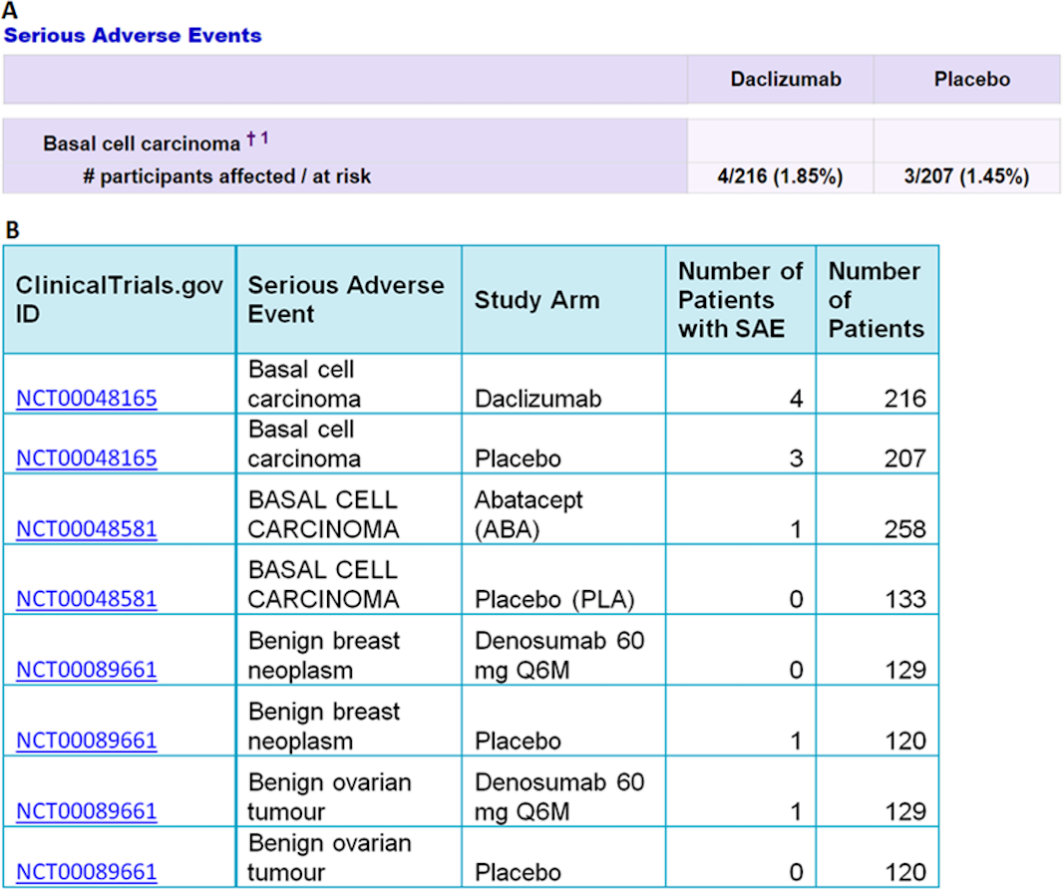
An example of the data extracted from ClinicalTrials.gov (A) into Excel (B) by the I2E query described above. The top two rows in (B) show the data extracted from the table in (A). The precision of the I2E query described above is 100%, and the recall is estimated as 99% assuming 1% of the cancer terms that the trial sponsors used are not among the cancer synonyms collected by MeSH or NCI.

The I2E query in Fig. 1 was run on the ClinicalTrails.gov index updated on August 14, 2016. The report contains 105,399 SAE events classified as cancer, from 2,861 randomized trials. An example of the extracted data is shown in Fig. 2.

The I2E output table was reformatted as illustrated in Table 1 to have one row per trial per SAE (type of cancer).

**Table 1:** A sample of the reformatted table.

ClinicalTrials.gov ID	Serious adverse event	Number of patients with SAE in control arm	Number of patients in control arm	Control arm	Number of patients with SAE in drug arm	Number of patients in drug arm	Drug arm
NCT00089791	Bladder cancer	3	3,876	Placebo	4	3,886	Denosumab 60 mg Q6M
NCT00089791	Breast cancer	25	3,876	Placebo	34	3,886	Denosumab 60 mg Q6M
NCT00089791	Colon cancer	8	3,876	Placebo	11	3,886	Denosumab 60 mg Q6M
NCT00120289	Lung neoplasm malignant	14	1,696	Placebo + Simvastatin	8	1,718	ERN + Simvastatin
NCT00120289	Malignant melanoma	4	1,696	Placebo + Simvastatin	1	1,718	ERN + Simvastatin
NCT00120289	Non-small cell lung cancer	4	1,696	Placebo + Simvastatin	0.3	1,718	ERN + Simvastatin
NCT00143507	Colon cancer	7	5,430	Placebo	5	5,477	Ivabradine
NCT00143507	Rectal cancer	6	5,430	Placebo	3	5,477	Ivabradine

If a row has less than 3 patients with SAE in the control arm, it is deleted. This is because the goal is to find drugs that have fewer cancer SAEs in the drug arm than in the control arm. After the deletions, the table has only 601 rows left.

If a row has 0 patients with SAE in the drug arm, the 0 value is replaced with 0.3. These replacements enable the ranking of the drugs that have no cancer SAE in the drug arm. Without the replacements, all such rows will have zero for OR and minus infinity for the *z*-score.

The final table with calculated columns is shown in Table 2. The drugs were ranked by sorting the *z*-score from the lowest value to the highest.

**Table 2:** The final table with calculated columns. The rows are sorted by z-score. Only the top 6 rows are shown (see Supplemental Information 2 for all 162 rows with *z* <  − 1).

Drug	Serious adverse event	*Ds*	*Dn*	*Cs*	*Cn*	Control	SE	OR	Lower limit	Upper limit	*z*	Clinical Trials.gov ID
V501	Cervical dysplasia	20	480	46	468	Placebo	0.28	0.40	0.23	0.69	−3.33	NCT00378560
Clopidogrel/ Telmisartan	Colon cancer	4	5,000	14	5,023	Clopidogrel/ Placebo	0.57	0.29	0.09	0.87	−2.20	NCT00153062
Vorapaxar	RECTAL CANCER	4	13,186	13	13,166	Placebo	0.57	0.31	0.10	0.94	−2.06	NCT00526474
Phylloquinone	Cancer	3	217	11	223	Placebo	0.66	0.27	0.07	0.98	−1.99	NCT00150969
Clopidogrel + ASA	Pancreatic carcinoma	1	3,772	8	3,782	Placebo + ASA	1.06	0.13	0.02	1.00	−1.96	NCT00249873
Core-phase: Aliskiren	Gastric cancer	1	4,272	8	4,285	Core-phase: Placebo	1.06	0.13	0.02	1.00	−1.96	NCT00549757

**Notes.**

TITLE *Ds* Number of patients with **S**AE in **D**rug arm *Dn* **N**umber of patients in **D**rug arm *Cs* Number of patients with **S**AE in **C**ontrol arm *Cn* **N**umber of patients in **C**ontrol arm

The original indications of the trials were (from top to bottom): HPV Infections, Stroke, Atherosclerosis, Osteoporosis, Atrial Fibrillation, and Type 2 Diabetes.

The results in Table 2 could range from false positive findings to possible signals for drug repositioning hypotheses. Therefore, we evaluated the drugs for cancer by other research from the current biomedical literature.

The V501 vaccine (Table 2, Row 1) arm had less cervical dysplasia events than the control in a clinical trial on the prevention of papillomavirus infection. Papillomavirus is already known to be associated with cervical dysplasia (Firnhaber et al., 2009), a precursor lesion of cancer of the cervix (Kesic, Petkovic & Milacic, 1990). We consider this top hit as a positive control that supports the credibility of our approach, since the prevention of the viral infection would naturally lead to the prevention of cervical dysplasia.

The data in Table 2, Row 2 suggest that Telmisartan might be useful to prevent colon cancer (note that Clopidogrel is in both the Drug and Control arm, so we did not investigate Clopidogrel further). Recent cell-based studies reported that Telmisartan exerts anti-tumor effects by activating peroxisome proliferator-activated receptor-*γ* (Li et al., 2014; Pu, Zhu & Kong, 2016; Wu et al., 2016b). The algorithm presented here provides the first evidence from a randomized clinical trial indicating that Telmisartan may be viable as a repurposed prevention for colon cancer.

Phylloquinone (Table 2, Row 4) is a vitamin (vitamin K1) supplement rather than a prescription drug. K vitamins + sorafenib induce apoptosis in human pancreatic cancer cell lines (Wei, Wang & Carr, 2010). A prospective cohort analysis found that individuals who increased their intake of dietary phylloquinone might have a lower risk of cancer than those who did not (Juanola-Falgarona et al., 2014). The data from the randomized trial in Table 2 suggest that vitamin K1 might actually help prevent cancer (*OR* = 0.27, 95% CI [0.07–0.98]). The potential cancer prevention by vitamin K1 is especially intriguing because one can get more than 1,000% daily value of vitamin K1 by simply eating one cup of cooked kale or spinach (https://www.healthaliciousness.com/articles/food-sources-of-vitamin-k.php).

The clinical trial in Table 2, row 6, tested Aliskiren for cardiovascular and renal disease in patients with type 2 diabetes. The SAE data from this study show that only 1 out of 4,272 patients in the Aliskiren arm reported gastric cancer versus 8 out of 4,285 patients in the placebo arm. A recent paper described that Aliskiren inhibits renal carcinoma cell lines proliferation *in vitro* (Hu et al., 2015). The data from this randomized clinical trial suggest the possible repurposing of Aliskiren for cancer.

Lastly, our literature search found no direct link between Vorapaxar (Table 2, Row 3) or Clopidogrel (Table 2, Row 5) and cancer prevention or treatment. Thus, these data in Table 2 could be the first sign that Vorapaxar or Clopidogrel might be useful for cancer or could be interpreted as false positive findings since we have made no attempt to adjust the multiplicity (multiple comparisons) in this exploratory analysis.

Above are only six outputs from our repositioning algorithm for one type of disease. The method described here could be used to identify other candidates for repositioning on any diseases that are reported as serious adverse events in ClinicalTrials.gov.

## Discussion

Presented here is a novel drug repositioning method that reveals potential new uses of existing drugs directly from clinical trial data. This article provides only a rudimentary way to conduct drug repositioning using text mining tools on ClinicalTrials.gov. However, it could serve to stimulate other investigational initiatives to use clinical data to repurpose drugs, supplements, or even food to help prevent or treat diseases.

Serious adverse event data from randomized trials in the ClinicalTrials.gov were used because randomized trials are controlled experiments. However, ClinicalTrials.gov is only a tiny part of clinical data that could lead to the discovery of new use of existing drugs. Electronic medical record databases have much more clinical data than ClinicalTrials.gov. Other large sources of clinical data include the Federal Adverse Event Reporting System and social media (Nugent, Plachouras & Leidner, 2016). These data could provide new information not only on marketed drugs, but also on supplements and food.

Computational drug repositioning usually involves the vast genome data and sophisticated machine learning techniques (Li et al., 2016). In contrast, the work described here uses relatively small clinical trial data on ClinicalTrials.gov, which has been proved useful in other works to identify combination therapy (Wu et al., 2015) and pharmacogenomics information (Li & Lu, 2012). The algorithm presented here is simple and direct. Combining this work with text mining (Tari & Patel, 2014) may lead to better methodologies for drug repurposing.

Compared to traditional drug development, repositioned drugs have the advantage of decreased development time and costs given that significant toxicology and safety data will have already been accumulated, drastically reducing the risk of attrition during the drug discovery and development process.

## Conclusions

The rapidly growing clinical data could be extracted and analyzed for drug repositioning utilizing text mining tools. Repositioning non-cancer drugs with low toxicity or even vitamin supplements for cancer might provide tangible benefits for patients.

The method described could be used for drug repositioning not only for cancer but also for other diseases and symptoms reported as adverse events. It might help other investigators to develop better ways to utilize the fast growing data in ClinicalTrials.com to reposition drugs for unmet medical needs.

The work we described here could merely help identify possible new uses of existing drugs to be investigated further. Prospective clinical trials would be required to provide the necessary evidence to have such new uses approved by regulatory agencies.

##  Supplemental Information

10.7717/peerj.3154/supp-1 Supplemental Information 1 Supplementary information on codes Instructions on how to replicate the I2E query in Fig. 1

10.7717/peerj.3154/supp-2 Supplemental Information 2 Expanded Table 3 With all results with *z*-score < − 1

## Additional Information and Declarations

### Data Availability

The following information was supplied regarding data availability: The raw data has been supplied as a Supplementary File.

## References

[ref-1] Ashburn TT, Thor KB (2004). Drug repositioning: identifying and developing new uses for existing drugs. Nature Reviews Drug Discovery.

[ref-2] Bandy J, Milward D, McQuay S (2009). Mining protein-protein interactions from published literature using Linguamatics I2E. Methods in Molecular Biology.

[ref-3] Coelho ED, Arrais JP, Oliveira JL (2016). Computational discovery of putative leads for drug repositioning through drug-target interaction prediction. PLOS Computational Biology.

[ref-4] Cormack J, Nath C, Milward D, Raja K, Jonnalagadda SR (2015). Agile text mining for the 2014 i2b2/UTHealth Cardiac risk factors challenge. Journal of Biomedical Informatics.

[ref-5] Firnhaber C, Zungu K, Levin S, Michelow P, Montaner LJ, McPhail P, Williamson AL, Allan BR, Van der Horst C, Rinas A, Sanne I (2009). Diverse and high prevalence of human papillomavirus associated with a significant high rate of cervical dysplasia in human immunodeficiency virus-infected women in Johannesburg, South Africa. Acta Cytologica.

[ref-6] Galijatovic-Idrizbegovic A, Miller JE, Cornell WD, Butler JA, Wollenberg GK, Sistare FD, DeGeorge JJ (2016). Role of chronic toxicology studies in revealing new toxicities. Regulatory Toxicology and Pharmacology.

[ref-7] Hodos RA, Kidd BA, Shameer K, Readhead BP, Dudley JT (2016). In silico methods for drug repurposing and pharmacology. Wiley Interdisciplinary Reviews: Systems Biology and Medicine.

[ref-8] Hu J, Zhang LC, Song X, Lu JR, Jin Z (2015). KRT6 interacting with notch1 contributes to progression of renal cell carcinoma, and aliskiren inhibits renal carcinoma cell lines proliferation *in vitro*. International Journal of Clinical and Experimental Pathology.

[ref-9] Juanola-Falgarona M, Salas-Salvado J, Martinez-Gonzalez MA, Corella D, Estruch R, Ros E, Fito M, Aros F, Gomez-Gracia E, Fiol M, Lapetra J, Basora J, Lamuela-Raventos RM, Serra-Majem L, Pinto X, Munoz MA, Ruiz-Gutierrez V, Fernandez-Ballart J, Bullo M (2014). Dietary intake of vitamin K is inversely associated with mortality risk. Journal of Nutrition.

[ref-10] Kesic V, Petkovic S, Milacic D (1990). Smoking and nonmalignant changes in the uterine cervix. Srpski Arhiv Za Celokupno Lekarstvo.

[ref-11] Li J, Chen L, Yu P, Liu B, Zhu J, Yang Y (2014). Telmisartan exerts anti-tumor effects by activating peroxisome proliferator-activated receptor-gamma in human lung adenocarcinoma A549 cells. Molecules.

[ref-12] Li J, Lu Z (2012). Systematic identification of pharmacogenomics information from clinical trials. Journal of Biomedical Informatics.

[ref-13] Li J, Zheng S, Chen B, Butte AJ, Swamidass SJ, Lu Z (2016). A survey of current trends in computational drug repositioning. Briefings in Bioinformatics.

[ref-14] Mullen J, Cockell SJ, Tipney H, Woollard PM, Wipat A (2016). Mining integrated semantic networks for drug repositioning opportunities. PeerJ.

[ref-15] Nugent T, Plachouras V, Leidner JL (2016). Computational drug repositioning based on side-effects mined from social media. PeerJ Computer Science.

[ref-16] Pu Z, Zhu M, Kong F (2016). Telmisartan prevents proliferation and promotes apoptosis of human ovarian cancer cells through upregulating PPARgamma and downregulating MMP9 expression. Molecular Medicine Reports.

[ref-17] Qosa H, Mohamed LA, Al Rihani SB, Batarseh YS, Duong QV, Keller JN, Kaddoumi A (2016). High-throughput screening for identification of blood-brain barrier integrity enhancers: a drug repurposing opportunity to rectify vascular amyloid toxicity. Journal of Alzheimer’s Disease.

[ref-18] Sheskin J (1965). Thalidomide in the treatment of lepra reactions. Clinical Pharmacology and Therapeutics.

[ref-19] Tari LB, Patel JH (2014). Systematic drug repurposing through text mining. Methods in Molecular Biology.

[ref-20] Wei G, Wang M, Carr BI (2010). Sorafenib combined vitamin K induces apoptosis in human pancreatic cancer cell lines through RAF/MEK/ERK and c-Jun NH2-terminal kinase pathways. Journal of Cellular Physiology.

[ref-21] Wu CH, Bai LY, Tsai MH, Chu PC, Chiu CF, Chen MY, Chiu SJ, Chiang JH, Weng JR (2016a). Pharmacological exploitation of the phenothiazine antipsychotics to develop novel antitumor agents-A drug repurposing strategy. Scientific Reports.

[ref-22] Wu M, Sirota M, Butte AJ, Chen B (2015). Characteristics of drug combination therapy in oncology by analyzing clinical trial data on ClinicalTrials.gov. Pacific Symposium on Biocomputing.

[ref-23] Wu TT, Niu HS, Chen LJ, Cheng JT, Tong YC (2016b). Increase of human prostate cancer cell (DU145) apoptosis by telmisartan through PPAR-delta pathway. European Journal of Pharmacology.

[ref-24] Zheng C, Guo Z, Huang C, Wu Z, Li Y, Chen X, Fu Y, Ru J, Ali Shar P, Wang Y, Wang Y (2015). Large-scale direct targeting for drug repositioning and discovery. Scientific Reports.

