# Supplementary material for: Systematic drug repositioning through mining adverse event data in ClinicalTrials.gov
Source: PeerJ. 2017 Mar 23;5:e3154. doi: 10.7717/peerj.3154 (PMC5366063; doi:10.7717/peerj.3154)
Supplement: Supplemental Information 1 — Instructions on how to replicate the I2E query in Fig. 1 [file peerj-05-3154-s001.docx]

**Systematic Drug Repositioning through Mining Adverse Event Data in ClinicalTrials.gov**

**Supplemental information**

**Supplementary information**

1. The YAML script of the I2E query in Figure 1:

To generate the query in Figure 1, copy and paste the YAML script below into the I2E Pro interface, remove query limits for hits, time, and rows, and add the “Same PTs” filter in the “Output editor” as shown in 2 below.

# Options: omitDefaults

# Written: Tue, 16 Aug 2016 21:10:33.898 GMT by I2E 4.4R31

---

version: 4.4

title: Extracting Serious Adverse Events

creationDate: '2015-07-30 11:31:14'

query:

document:

- region:

showInColumn: false

showInHitColumn: false

snid: ct.event_se

pt: Serious Event

where:

- region:

columnOptions: [Text]

id: region2

view: {height: 87, width: 165}

snid: ct.sub_title_se

pt: Serious Event Subtitle

where:

- class: {showInColumn: false, showInHitColumn: false, snid: nlm_plus.C04,

pt: Neoplasms}

- region:

columnOptions: [Text]

showInColumn: false

showInHitColumn: false

snid: ct.counts_se

pt: Serious Event Counts

where:

- region:

showInColumn: false

snid: ct.group_id

pt: Group IDs

where:

- class: {showInColumn: false, showInHitColumn: false, parameterisationEnabled: false,

id: class3, snid: /word}

- region:

columnOptions: [Text]

id: region4

view: {height: 42, width: 300}

snid: ct.subjects_affected_se

pt: Count of Participants Affected by Serious Event

- region:

columnOptions: [Text]

id: region5

view: {height: 44, width: 298}

snid: ct.subjects_at_risk_se

pt: Count of Participants At Risk of Serious Event

- region:

view: {height: 129, width: 289}

snid: ct.re_group

pt: Reported Adverse Events Reporting Group

where:

- region:

showInColumn: false

snid: ct.group_id

pt: Group IDs

where:

- class: {showInColumn: false, showInHitColumn: false, parameterisationEnabled: false,

id: class2, snid: /word}

- region:

columnOptions: [Text]

id: region3

snid: ct.title

pt: Title

- region:

identifiers:

- {snid: ct.study_design, pt: Study Design}

- {snid: ct.official_title, pt: Official Title}

view: {height: 96, width: 197}

where:

- word: {text: random*, id: word1, matchType: Wildcard, showInColumn: false}

- region:

snid: ct.condition

pt: Condition

where:

- class: {role: negated, showInColumn: false, showInHitColumn: false, parameterisationEnabled: false,

snid: nlm_plus.C04, pt: Neoplasms}

output:

columns:

- {id: region2}

- {title: Study Arm, id: region3}

- {id: class2}

- {title: Num. of Ptnts w SAE, id: region4}

- {title: Number of Patients, id: region5}

- {id: class3}

- {id: word1}

outputSettings:

allDocs: true

allResults: true

allRows: false

allTime: true

boundaries: Default

crossProduct: true

defaultColumnOptions: [Default]

documentsPerAssertion: -1

fileFormatValue: dhtml

globalDisambiguationValue: 12

hitsPerDoc: 10000

hitsPerDocPerAssertion: 10

language: mul

maxDocs: 10000

maxResults: 1000

maxRows: 110000

maxTime: 60

outputOrdering: frequency

outputType: cluster

overrideDisambiguation: false

resultType: standard

showQueryIDs: true

useOutputEditor: true

filters:

- of: PT

where: [class2, class3]

condition: equals

comments: "Please first double-click \"CTgov.i2x\" on the upper left window to open\

\ the index (database).\n\nTo select type of disease, go to Query editor and double-click\

\ the two disease folders (Neoplasms, do one at a time), click the upper level \n\

folder \"Disease\", then click \"More\" button and select \"Below selection\", type\

\ in your disease term next to \"Look for\" and enter\nor click \"Search\" (may\

\ take a minute, click \"Stop\" when a good hit is found), click the correct term\

\ in the results, click OK.\n\nTo run your query, click the green arrow on top right.\

\ When finished, click the refresh button in the new web browser\n(the two green\

\ arrows like a circle) to retrieve all the hits (may take a minute).\n\nTo view\

\ the results, click the triangles to see more, and then click a term under \"Hit\"\

\ to see the document with query terms highlighted.\n\nTo export to the Excel, change\

\ \"HTML\" in the result (browser) window upper right to \"Excel\", then click the\

\ green arrow, click \"Open\", \"Yes\"\n(If no dialog window for \"Yes\" shows up,\

\ minimize windows to see it behind them).\n\nPlease contact Eric Su (ewsu@lilly.com,\

\ 317-277-7706) for questions.\n\nÂ© 2016 Eli Lilly and Company\n"

creator: Eric Su

summary: This query extracts serious adverse events from clinicaltrials.com.

useInSmartQuery: true

1. Output editor setting of the I2E query in Figure 1:


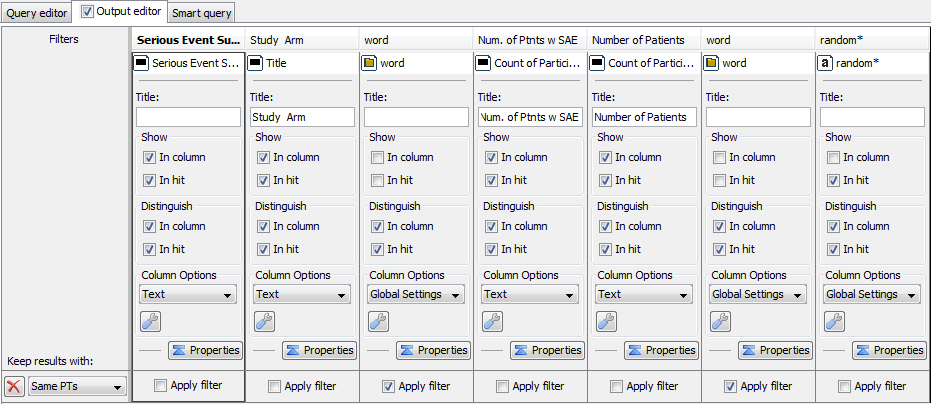


The “word” classes inside Group IDs in Figure 1 are matched with the “Same PTs” (the same preferred term) to match the data to the reporting groups (study arms).

1. PolyAnalyst script will be also available upon request to EWS. However, the algorithm for calculating the statistics columns in Table 3 could also be implemented by SAS or one of the open-source packages such as KNIME, R or Python.
